# Exceptional response to lorlatinib and cabozantinib in ROS1-rearranged NSCLC with acquired F2004V and L2086F resistance

**DOI:** 10.1038/s41698-023-00381-0

**Published:** 2023-06-08

**Authors:** Mandy Sakamoto, Tejas Patil

**Affiliations:** Department of Medicine, Division of Medical Oncology, University of Colorado School of Medicine, Aurora, CO USA

**Keywords:** Non-small-cell lung cancer, Molecular medicine

## Abstract

Patients with ROS1-rearranged NSCLC demonstrate excellent disease control with ROS1-targeted therapy, but acquired resistance is inevitable. Of particular interest is the ROS1 L2086F kinase domain mutation which is refractory to all currently available ROS1 TKIs apart from cabozantinib. We present a case of a patient with metastatic ROS1-rearranged NSCLC with dual ROS1 F2004V and L2086F resistance mutations who radiographically responded to the combination of lorlatinib and cabozantinib in a patient with metastatic NSCLC. Furthermore, the patient experienced exceptional clinical improvement and tolerance with the combined use of lorlatinib and cabozantinib. This case builds the case for cabozantinib as an agent to overcome ROS1 L2086F resistance. It also highlights the efficacy and safety of using combination of ROS1 TKIs to overcome complex resistance patterns.

## Introduction

Chromosomal rearrangements involving the ROS1 receptor tyrosine kinase gene represent a unique subset of molecular drivers seen in ~1–2% of all non-small cell lung cancers (NSCLCs)^1^. ROS1-rearranged NSCLC is highly sensitive to tyrosine kinase inhibitors (TKI), with superior response rates and clinical outcomes compared to platinum doublet chemotherapy^2–4^. On-target resistance mutations (such as G2032R and L2026M) invariably develop and represent a major clinical challenge. Newer generation ROS1 TKIs are in development to address gatekeeper, solvent front, and dual kinase domain mutations. However, L2086F has emerged a problematic kinase domain resistance mutation that is resistant to all known ROS1 TKIs with the exception of cabozantinib^5–8^. Here we report a case of a heavily pre-treated patient with stage IV ROS1-rearranged NSCLC with dual ROS1 F2004V and L2086F kinase domain resistance mutations who had an excellent clinical response to the combination of lorlatinib and cabozantinib.

## Results

### Case

A 22-year-old woman with no smoking history was found to have an amelanotic choroidal tumor after presenting with several months of visual symptoms. Subsequent positron emission tomography/computed tomography (PET/CT) revealed multiple fluorodeoxyglucose (FDG)-avid lung nodules and widespread osseous metastases. A CT-guided lung biopsy demonstrated moderately differentiated adenocarcinoma with lepidic features. Magnetic resonance imaging (MRI) of the brain was negative for intracranial metastases. Molecular testing using next-generation sequencing (NGS) assay identified a *CD74-ROS1* rearrangement. The patient completed intensity modulated radiation therapy (IMRT) to the right retina and received crizotinib 250 mg twice daily. She achieved a complete radiographic response until 10 months later when her brain MRI showed numerous small enhancing lesions consistent with isolated CNS progression. She was started on entrectinib 600 mg daily as part of the STARTRK-1 phase 1/2a clinical trial with stable disease until seven months later when she developed asymptomatic progression in the brain. She received stereotactic radiosurgery (SRS) and entrectinib was dose escalated to 800 mg daily. She remained on this increased dose of entrectinib with good tolerance for over two years during which time she again progressed in the CNS requiring additional treatments with SRS, but otherwise had stable disease in the body. She eventually developed new osseous metastases. Molecular testing using the circulating tumor DNA (ctDNA) NGS assay identified a ROS1 F2004V kinase domain resistance mutation. She received lorlatinib 100 mg daily. After 10 months, the decision was made to start chemotherapy with carboplatin (AUC 6) and pemetrexed 500 mg/m^2^ in combination with lorlatinib due to further osseous progression. She required frequent dose interruptions of pemetrexed due to renal insufficiency and cytopenias. Pemetrexed was also thought to cause a type 3 hypersensitivity reaction manifesting as rash, fevers, and peripheral eosinophilia.

**Fig. 1:**
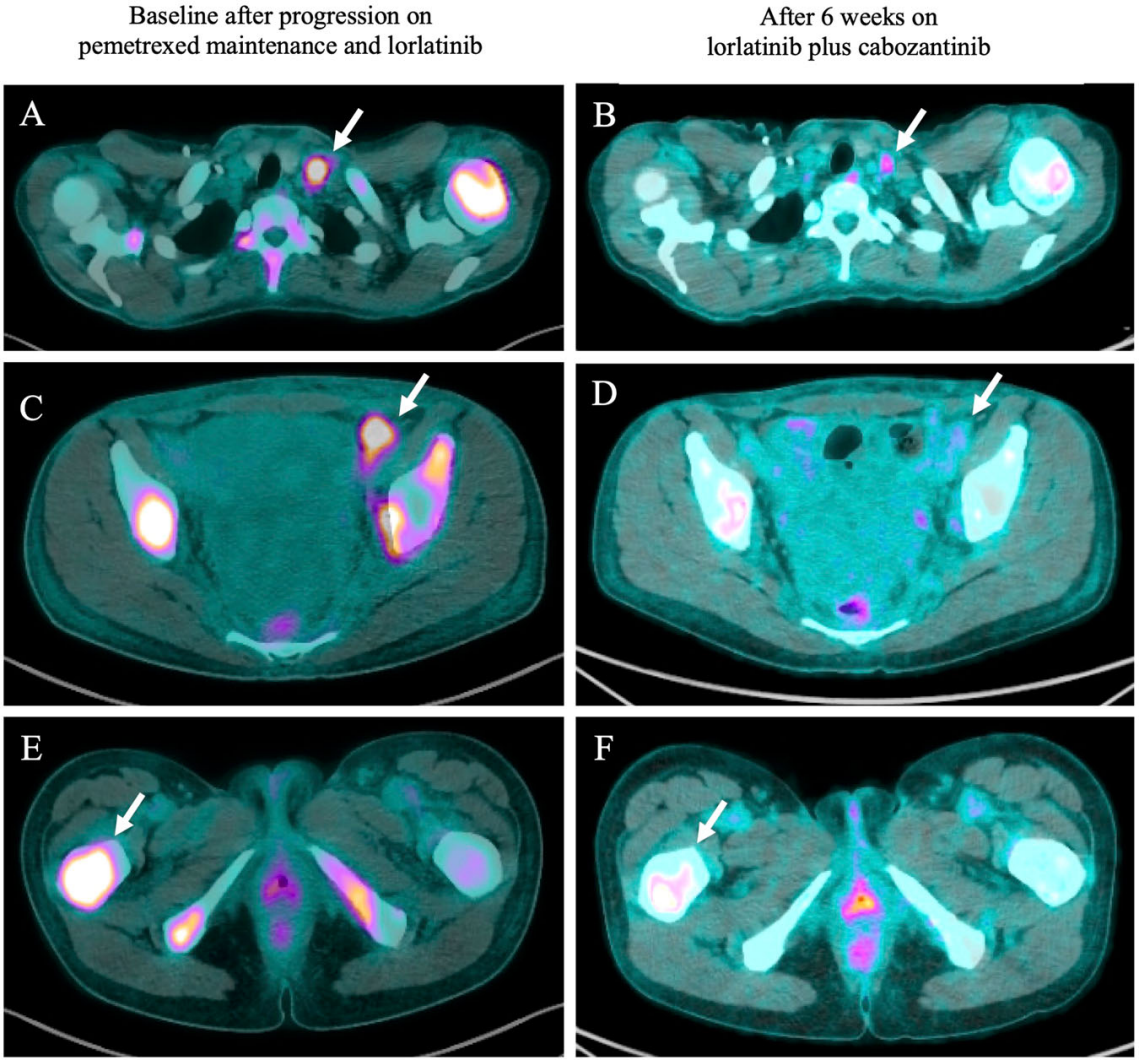
Fused PET/CT imaging illustrating combination lorlatinib and cabozantinib mediated response after development of a ROS1 L2086F resistance mutation. **A**, **B** Response in the left cervical lymph node including decreased size and significantly decreased FDG uptake with maximum SUV of 8.2 at baseline and at 6 weeks follow-up. **C**, **D** Near complete resolution of left external iliac nodes with maximum SUV of 6.9 at baseline and at 6 weeks follow-up. **E**, **F** Response in the right proximal femur including decreased size and FDG uptake with maximum SUV of 10.7 at baseline and 8.3 at 6 weeks follow-up. Multiple other metastatic osseous lesions and lymph nodes also demonstrate decreased size and uptake (not shown).

**Fig. 2:**
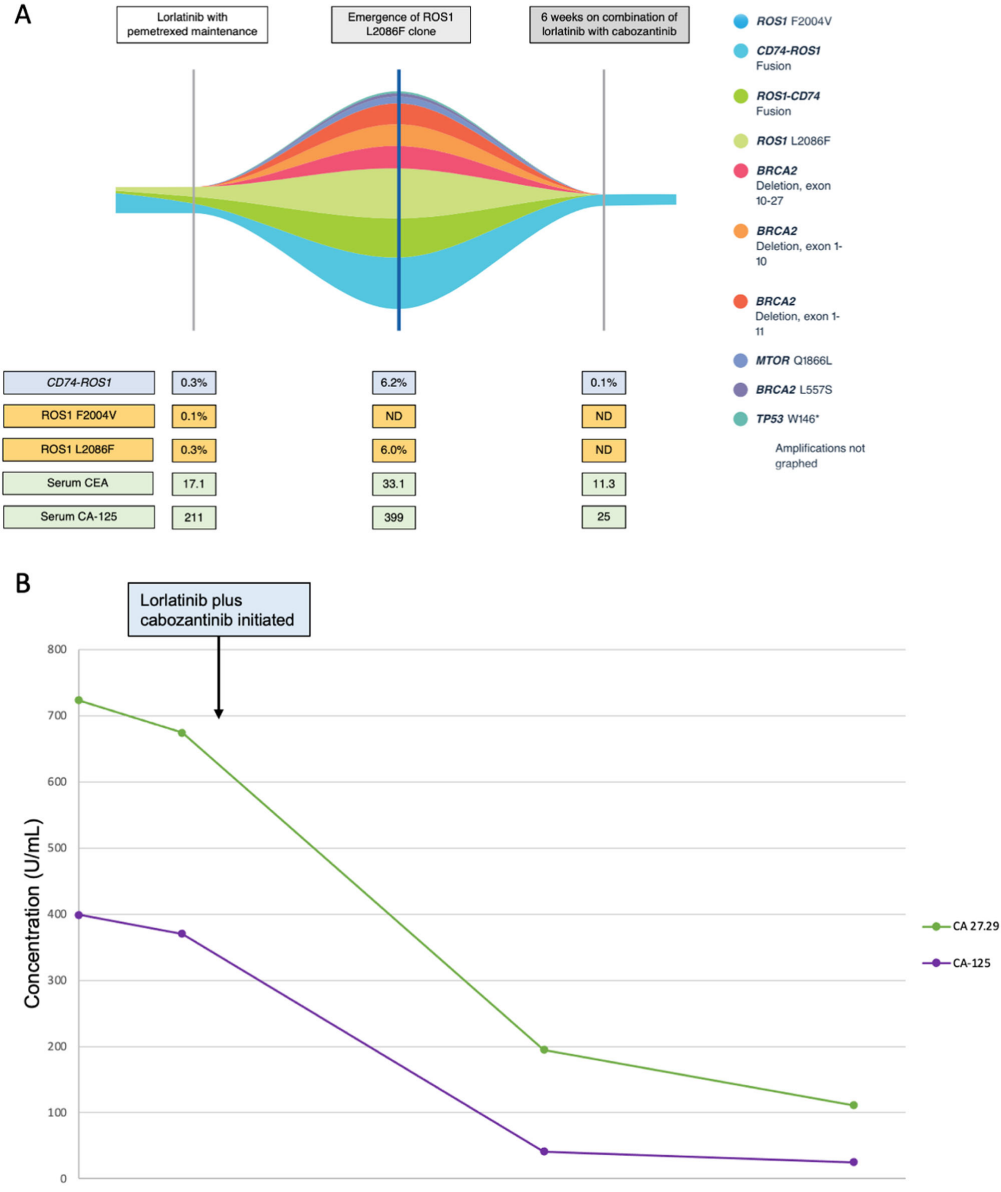
Molecular and serological response to dual TKI with lorlatinib plus cabozantinib. **A** Guardant® 360 circulating tumor DNA response map showing dynamic changes in variant allele fractions. **B** Changes in tumor markers CA 27.29 and CA-125 during treatment.

**Fig. 3:**
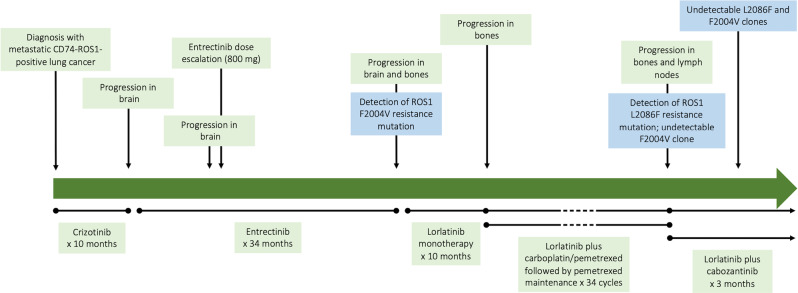
Timeline of clinical course including treatment duration and molecular data. A flow diagram detailing the clinical course of this patient.

After 34 cycles of pemetrexed, a PET/CT demonstrated widespread progression in bones and multiple cervical, axillary, and pelvic lymph nodes. On physical exam, she was found to have a 2 cm firm, palpable, non-tender cervical lymph nodes. Repeat molecular testing using ctDNA NGS identified a ROS1 L2086F resistance mutation and F2004V was undetectable. She was started on cabozantinib 40 mg daily in combination with lorlatinib 100 mg daily. She reported significant improvement in symptoms including resolution of cervical adenopathy and increase in energy level. Her first on-treatment PET/CT scan after 6 weeks of therapy showed improvement in FDG-avid osseous lesions and decreased size and avidity of lymphadenopathy (Fig. 1). Her MRI brain demonstrated no intracranial progression. Repeat ctDNA NGS testing at this time demonstrated an undetectable L2086F clone (Fig. 2). Four weeks after starting cabozantinib, she developed stage 1 hypertension that was well controlled on two antihypertensive agents (the first antihypertensive agent was started in the setting of renal insufficiency prior to initiation of cabozantinib). She reported intermittent grade 1 lightheadedness but otherwise tolerated the combination of lorlatinib and cabozantinib without additional adverse effects. There were no dose reductions or treatment interruptions required. Figure 3 illustrates the patient’s treatment history and from the time of diagnosis. At the time of this report, the patient has been on the combination of lorlatinib 100 mg and cabozantinib for a total of 11 months.

## Discussion

We describe a ROS1-fusion positive NSCLC patient with dual F2004V and L2086F resistance mutations overcome by the combination of lorlatinib plus cabozantinib, as evidenced by an impressive radiographic response and undetectable L2086F clone shortly after starting therapy. ROS1 L2086F is a novel kinase domain mutation that emerged as a mechanism of acquired resistance to lorlatinib and is known to be refractory to all currently available ROS1 TKIs with the exception of cabozantinib. To our knowledge, we are also the first to report excellent tolerance to the combination of cabozantinib and lorlatinib.

Lorlatinib is a highly potent, next-generation TKI currently recommended in advanced ROS1-rearranged NSCLC after disease progression on crizotinib, entrectinib, or ceritinib^9^. A recent study by Lin et al characterized mechanisms of resistance to lorlatinib in ROS1-positive tumors, many of which involve specific ROS1 kinase domain mutations with the most common being G2032R (32%) followed by L2086F (11%)^5^. ROS1 L2086F is caused by the substitution of leucine for a phenylalanine residue at amino acid 17. Structural modeling of the ROS1 L2086F mutant showed the resultant conformational change in the ROS1 receptor, leading to steric interference with the binding of lorlatinib and other type I TKIs^5^. Accordingly, tumors harboring this mutation were refractory to nearly all ROS1 TKIs, even at higher drug concentrations. Preclinical studies showed that lorlatinib, crizotinib, entrectinib, ceritinib, taletrectinib, and repotrectinib were not potent against this mutation, with half-maximal inhibitory concentrations (IC_50_) well over 200 nmol/L^5^. A lower IC_50_ value was seen with brigatinib (IC_50_ of 159.3 nmol/L) but was still 17-fold less potent as compared to ROS1 wild-type (IC_50_ of 9.4 nmol/L). Cabozantinib, a type II multi-kinase TKI, was the only agent that demonstrated strong potency against L2086F (as well as L2086F-containing compound mutants) with an IC_50_ of 3.6 nmol/L. Based on this data, a patient with an acquired ROS1 L2086F mutation after progression on lorlatinib was treated with cabozantinib monotherapy with disease control lasting almost 11 months^5^.

In this case, the decision to continue lorlatinib with cabozantinib was based on two observations. First, prior studies have suggested that substitutions of the ROS1 F2004 position are less sensitive to type II MET inhibitors, though are susceptible to certain type I MET inhibitors^10,11^. In particular, ROS1 F2004V mutations have been shown to be highly resistant to cabozantinib^11^. Given these findings, we elected to continue lorlatinib with cabozantinib. Cabozantinib alone has been used against ROS1 L2086F after progression on lorlatinib and taletrectinib^5,7^, but cabozantinib has never been explored in the context of addressing dual ROS1 on-target mutations. There are three plausible explanations for the low allelic frequency of F2004V prior to starting lorlatinib and cabozantinib. First, is that the ROS1 F2004V clone was appropriately suppressed by the continuation of lorlatinib. Second, F2004V subclone may have been eliminated by the effects of platinum doublet chemotherapy given the very low levels detected at the time of pemetrexed maintenance. Third, the ROS1 F2004V mutation may reflect a subclonal event occurring within the bone below the threshold detection limits of the ctDNA assay.

The second reason to continue lorlatinib with cabozantinib was that our patient had known intracranial metastases that were controlled with lorlatinib. The intracranial activity of cabozantinib in ROS1 NSCLC has only been described in a single case series of four ROS1-positive NSCLC patients with progression on crizotinib^12^. In this series, two out of four patients had intracranial disease prior to receiving cabozantinib with stable disease as best radiographic response for both patients. Among patients with metastatic renal cell carcinoma, the intracranial response rate of cabozantinib is 55%^13^.

Our findings may help guide sequential ROS1 TKI therapy with a potential role for type II TKIs such as cabozantinib in the setting of L2086F-mediated resistance. Ongoing efforts to develop novel, selective type II ROS1 inhibitors effective against ROS1 kinase domain resistance mutations are greatly needed. Finally, recognition of co-existing resistance mutations that are less responsive to type II TKIs is critical, as this may warrant a combination approach with both type I and type II ROS1 TKIs.

## Methods

### Patient

The patient provided written informed consent for blood collection, ctDNA analysis, tissue biopsies, treatment, and publication of this report.

### Clinical testing

Molecular testing was conducted by the CLIA certified Colorado Genetics Laboratory (CGL) and Colorado Molecular Correlates Laboratory (CMOCO) and performed using laboratory assays that were independently validated. Next-generation sequencing was performed using a customized version of the ArcherDx VariantPlex and FusionPlex Solid Tumor library preparation kit was used (ArcherDx, Boulder, CO). Libraries from this assay were sequenced on the Illumina platform, and raw sequence data were processed using the ArcherDx Analysis package v5.1.2. Libraries were sequenced on the Illumina platform, and raw sequence data were processed using the ArcherDx Analysis package v4.1.1.7. For ctDNA analysis, the Guardant® 360 assay (Illumina, San Diego, CA) was utilized using hg19 as the reference genome. For all research performed using patient data in this study appropriate approval was obtained and all ethical regulations related to the use of this data were followed.

### Reporting summary

Further information on research design is available in the Nature Research Reporting Summary linked to this article.

## Supplementary information

Reporting summary

## Data Availability

All the data and resources generated for this study are available in the article or from the corresponding author upon request. CGH array data are deposited in GEO under the accession number GSE180894.
